# Diagnostic value of cerebrospinal fluid tau, neurofilament, and progranulin in definite frontotemporal lobar degeneration

**DOI:** 10.1186/s13195-018-0364-0

**Published:** 2018-03-20

**Authors:** Joery Goossens, Maria Bjerke, Sara Van Mossevelde, Tobi Van den Bossche, Johan Goeman, Bart De Vil, Anne Sieben, Jean-Jacques Martin, Patrick Cras, Peter Paul De Deyn, Christine Van Broeckhoven, Julie van der Zee, Sebastiaan Engelborghs

**Affiliations:** 1 Reference Center for Biological Markers of Dementia, Laboratory of Neurochemistry and Behavior, University of Antwerp, Universiteitsplein 1, 2610 Wilrijk, Belgium; 2 Institute Born-Bunge, University of Antwerp, Universiteitsplein 1, 2610 Wilrijk, Belgium; 3 Neurodegenerative Brain Diseases Group, Center for Molecular Neurology, VIB, Universiteitsplein 1, 2610 Wilrijk, Belgium; 4 Department of Neurology and Memory Clinic, Hospital Network Antwerp (ZNA) Middelheim and Hoge Beuken, 2660 Antwerpen, Belgium; 5 Department of Neurology, Antwerp University Hospital, Wilrijkstraat 10, 2650 Edegem, Belgium; 6 Laboratory of Neurology, Translational Neurosciences, University of Antwerp, Universiteitsplein 1, 2610 Wilrijk, Belgium

**Keywords:** Frontotemporal lobar degeneration, Alzheimer’s disease, Cerebrospinal fluid, Biomarkers, Differential diagnosis, Tau, Neurofilament, Progranulin

## Abstract

**Background:**

We explored the diagnostic performance of cerebrospinal fluid (CSF) biomarkers in allowing differentiation between frontotemporal lobar degeneration (FTLD) and Alzheimer’s disease (AD), as well as between FTLD pathological subtypes.

**Methods:**

CSF levels of routine AD biomarkers (phosphorylated tau (p-tau_181_), total tau (t-tau), and amyloid-beta (Aβ)_1–42_) and neurofilament proteins, as well as progranulin levels in both CSF and serum were quantified in definite FTLD (*n* = 46), clinical AD (*n* = 45), and cognitively healthy controls (*n* = 20). FTLD subgroups were defined by genetic carrier status and/or postmortem neuropathological confirmation (FTLD-TDP: *n* = 34, including FTLD-*C9orf72*: *n* = 19 and FTLD-*GRN*: *n* = 9; FTLD-tau: *n* = 10).

**Results:**

*GRN* mutation carriers had significantly lower progranulin levels compared to other FTLD patients, AD, and controls. Both t-tau and p-tau_181_ were normal in FTLD patients, even in FTLD-tau. Aβ_1–42_ levels were very variable in FTLD. Neurofilament light chain (Nf-L) was significantly higher in FTLD compared with AD and controls. The reference logistic regression model based on the established AD biomarkers could be improved by the inclusion of CSF Nf-L, which was also important for the differentiation between FTLD and controls. Within the FTLD cohort, no significant differences were found between FTLD-TDP and FTLD-tau, but *GRN* mutation carriers had higher t-tau and Nf-L levels than *C9orf72* mutation carriers and FTLD-tau patients.

**Conclusions:**

There is an added value for Nf-L in the differential diagnosis of FTLD. Progranulin levels in CSF depend on mutation status, and *GRN* mutation carriers seem to be affected by more severe neurodegeneration.

## Background

Frontotemporal lobar degeneration (FTLD) is the primary cause of early-onset dementia after Alzheimer’s disease (AD) [1]. Major molecular pathologies underlying FTLD include aggregation of transactive response DNA-binding protein of 43 kDa (TDP-43, FTLD-TDP) or tau (FTLD-tau) [2, 3]. While each molecular pathology is associated with mutations in specific genes (e.g., *GRN*, *C9orf72*, *MAPT*) there is clinical overlap across pathologies and with AD [4–6]. Clinical diagnosis of both dementias is primarily based on exclusion of other diseases, and this results at best in a diagnosis of probable AD or probable FTLD [7–10]. Correct identification of FTLD and its associated pathology is of importance for protein-specific research and treatment (e.g., disease modifiers). Diagnostic accuracy for differentiation between FTLD and AD, as well as between FTLD pathological subtypes, could be increased by quantification of disease-specific biochemical markers present in biofluids (cerebrospinal fluid (CSF) and blood) [10, 11].

At present, well-characterized and validated diagnostic markers specific for FTLD pathology do not exist, with the exception of decreased progranulin concentrations in serum or plasma for *GRN* mutation-related FTLD, an important subgroup of FTLD-TDP [12, 13]. However, progranulin may play a pathophysiological role in the brain as well, independent of *GRN* mutations [14], and this might be reflected in progranulin levels in CSF which are shown to be mainly brain-derived and regulated independently from levels in the blood [15, 16]. On the other hand, one previous study has questioned the pathological role of progranulin by showing that CSF progranulin levels could not differentiate between clinically diagnosed groups of AD and FTLD patients [17].

As FTLD can also present with tau-positive inclusions as a primary pathology, the AD CSF biomarkers total tau (t-tau) and hyperphosphorylated tau at threonine 181 (p-tau_181_) are also interesting markers. In theory, an increased concentration of both could be expected since t-tau is an aspecific marker for neurodegeneration and p-tau_181_ is a marker of tau pathology [18]. However, in practice, mixed results have been generated over the years, with CSF levels of both biomarkers generally being intermediate in FTLD compared with AD and controls [19]. The combination of t-tau and p-tau_181_ with the third AD biomarker amyloid-beta of 42 amino acids (Aβ_1–42_) can at least aid in differential dementia diagnosis (reviewed in [20]). It is noteworthy that recent studies have shown that the p-tau_181_/t-tau ratio can be useful in the differentiation of FTLD-tau from FTLD-TDP [21, 22].

Next to pathology-specific biomarkers, other candidates have been proposed. Most promising are neurofilaments which are structural axonal proteins, and their presence in CSF is a marker for neurodegeneration [23]. These proteins might have a more specific role in FTLD-TDP, as TDP-43 interacts with neurofilament light chain (Nf-L) mRNA [24]. It has been reported that both Nf-L and (phosphorylated) neurofilament heavy chain (Nf-H) subunits can be detected in the CSF of FTLD patients, allowing differentiation of FTLD from AD and controls, and differentiation between FTLD subtypes [25, 26].

This study aimed to explore the performance of proven and candidate CSF biomarkers to improve differential diagnosis of FTLD. A secondary aim was to assess if CSF progranulin levels are dependent on mutation status only or are also related to FTLD pathology.

## Methods

### Study cohort

The study population consisted of definite FTLD patients (*n* = 46), defined by genetic carrier status and/or postmortem neuropathological confirmation [27, 28]. These patients could be subdivided into FTLD-TDP (*n* = 34, including 19 FTLD-*C9orf72*, 9 FTLD-*GRN*, 1 FTLD-*VCP*, and 1 FTLD-*TBK1* symptomatic mutation carriers; 19 pathologically confirmed), FTLD-tau (*n* = 10, including 1 FTLD-*MAPT* symptomatic mutation carrier; 9 pathologically confirmed), or FTLD-other (*n* = 2, both pathologically confirmed). Clinical diagnosis of definite FTLD patients consisted of the behavioral variant frontotemporal dementia (*n* = 30), AD (*n* = 7), primary progressive aphasia (*n* = 3), progressive supranuclear palsy (*n* = 2), corticobasal degeneration (*n* = 1), and other types of dementia (*n* = 3). AD patients (*n* = 45) were clinically diagnosed based on IWG-2 criteria, including the AD CSF biomarker panel (pathological cut-offs: Aβ_1–42_ < 638.5 pg/mL, t-tau > 296.5 pg/mL, p-tau_181_ > 56.5 pg/mL) [10, 29]. Together with extensive clinical follow-up (median 4.9 (range 2.7–7.9) years) this added to the diagnostic certainty. The control group (*n* = 20) consisted of elderly people who had no neurological or psychiatric antecedents and no organic disease involving the central nervous system following extensive clinical examination (patients with polyneuropathy, *n* = 8; patients with subjective complaints, *n* = 12). All CSF samples (full cohorts) and available blood samples (controls, *n* = 18; AD, *n* = 42; FTLD, *n* = 30) were selected from the Institute Born-Bunge (IBB) Biobank, Antwerp, Belgium [30]. Data including gender, Mini-Mental State Examination (MMSE) score, age at time of CSF sampling, age at onset, and age at death (if applicable) were available for the majority of the patients. This study was approved by the ethics committee of the University of Antwerp, Antwerp, Belgium (B300201420405).

### Biomarker analysis

Lumbar puncture (LP), and CSF and blood sampling and handling was performed according to a standardized protocol [30, 31]. All CSF and blood samples were stored at the IBB Biobank in polypropylene vials at −80 °C until analysis.

CSF biomarker levels were quantified using commercially available single-analyte enzyme-linked immunosorbent assay (ELISA) kits (one kit lot each), strictly following the manufacturer’s instructions (INNOTEST β-Amyloid(_1–42_), INNOTEST hTau-Ag, and INNOTEST Phospho-Tau(_181P_) from Fujirebio Europe, Belgium; Nf-light from UmanDiagnostics, IBL International GmbH, Germany; pNF-H V2 from EnCor Biotechnology Inc., USA; human progranulin from Adipogen Inc., Korea). The last kit was also used to quantify serum levels of progranulin. All samples were run in duplicate, blinded for diagnosis. Intra-assay coefficient of variation was below 10% for all analytes.

### Statistical analysis

Statistical testing was performed using IBM SPSS Statistics 23, GraphPad Prism 6, and the R package ‘pROC’ in RStudio (version 1.0.136) [32]. As some variables were not normally distributed and numbers in FTLD subgroups were small, nonparametric analyses were used. Kruskal-Wallis analyses were performed to describe the dementia patient cohorts and compare biomarker levels between groups. Post-hoc analyses included Dunn’s correction for multiple comparisons. For pairwise comparisons between FTLD pathological and genetic subgroups, Mann-Whitney *U* tests were performed. Categorical variables were analyzed with a Chi-square test. Spearman’s ρ was calculated to determine correlations. Logistic regression models were generated using a forward selection method of AD biomarkers alone, or of all available single CSF biomarkers. For these analyses, biomarker data were log_10_-transformed to achieve normality. Receiver operating characteristic (ROC) curve analyses were used to obtain area under the curve (AUC) values with 95% confidence intervals (CIs) for differentiation between groups [32, 33]. AUC values were compared using DeLong tests. The maximal sum of sensitivity and specificity (maximized Youden’s index) was calculated to determine cut-off values for progranulin. For all analyses, *p* values below 0.05 were considered statistically significant.

## Results

### Biomarker results

Demographic, clinical, and biomarker data for all groups are summarized in Table 1. Two FTLD patients were excluded from the statistical analysis as none of their AD biomarker values could be determined, probably related to preanalytical factors. Biomarker values outside of the assay limits of detection were set to the lowest/highest detection point ±20%, and this value was used in nonparametric statistical analysis (t-tau: 1 FTLD and 5 AD patients; p-tau_181_: 3 FTLD patients; Nf-L: 3 FTLD patients, 2 controls, and 3 AD patients; pNf-H: 9 FTLD, 7 controls, and 8 AD patients). There was no difference in biomarker levels between males and females (*p* = 0.53). Age at LP correlated significantly with Nf-L levels in controls and AD patients, but not in the FTLD group (controls: ρ = 0.837, *p* < 0.001; AD: ρ = 0.415, *p* < 0.01; FTLD: ρ = 0.006, *p* = 0.97). MMSE score correlated with Aβ_1–42_ (0.436, *p* < 0.01) and Nf-L (−0.457, *p* < 0.01) levels in AD patients. No notable correlations were found for the different clinical features and biomarker levels in the FTLD group or its subgroups.

**Table 1:** Demographic information and biomarker data

		FTLD subgroups					
				FTLD-TDP subgroups			
	FTLD	FTLD-tau	FTLD-TDP	FTLD-*C9orf72*	FTLD-*GRN*	Controls	Alzheimer’s disease
Gender, % male/female (*n*)	50/50 (46)	70/30 (10)	44/56 (34)	37/63 (19)	56/44 (9)	55/45 (20)	49/51 (45)
Age at LP (years)	63.6 (55.1–71.7)^a^	70.3 (56.2–74.4)	63.3 (54.5–71.6)	59.0 (53.7–69.5)*	67.3 (63.3–71.9)	69.4 (61.5–74.7)	71.2 (66.7–79.2)
Age at onset (years)	62.0 (53.8–69.2)^a^	56.0 (54.0–71.0)	63.5 (52.8–69.2)	56.0 (48.0–67.0)	66.0 (61.5–70.0)	N/A	69.5 (64.3–75.8)
Age at death (years)	65.9 (60.2–75.1)	74.0 (60.8–76.2)	62.9 (57.5–72.5)	57.7 (52.3–62.6)*^†^	69.1 (61.1–74.2)	N/A	N/A
*APOE* ε4 carriers, % (*n*)	32.4 (37)	57.1 (7)	27.6 (29)	30.8 (13)	20.0 (10)	33.3 (6)	59.5 (42)
MMSE at LP (0–30), (*n*)	21 (15–25) (29)	23 (22–27) (5)	19 (14–25) (23)	15 (7–21) (10)^†^	23 (16–26) (7)	N/A	20 (15–25) (42)
Serum progranulin (ng/mL)	95 (60–126)^a,b^	96 (76–167)*	95 (61–125)	107 (91–125)*	48 (39–63)	130 (101–175)	119 (98–145)
CSF progranulin (ng/mL)	3.39 (2.29–3.85)^a^	3.79 (2.94–4.23)*	3.00 (2.10–3.64)	3.21 (2.35–3.74)*	1.93 (0.97–2.43)	3.61 (2.92–4.50)	3.88 (3.22–4.59)
CSF Aβ_1–42_ (pg/mL)	641 (457–858)^a^	543 (438–960)	698 (481–862)	695 (457–819)	708 (578–943)	812 (646–1108)	509 (372–594)^b^
CSF t-tau (pg/mL)	320 (219–420)^a^	331 (197–400)	330 (236–464)	252 (178–332)*	379 (296–559)	257 (173–381)	627 (429–928)^b^
CSF p-tau_181_ (pg/mL)	36.7 (28.3–49.0)^a^	35.5 (26.9–58.8)	37.2 (28.8–49.0)	33.0 (22.0–49.0)	36.0 (30.9–42.5)	40.3 (32.9–58.6)	80.0 (60.5–105.0)^b^
CSF p-tau_181_/t-tau ratio	0.117 (0.097–0.149)^b^	0.144 (0.095–0.152)*	0.116 (0.092–0.145)	0.142 (0.105–0.164)*	0.088 (0.076–0.114)	0.176 (0.156–0.197)	0.132 (0.104–0.149)^b^
CSF Nf-L (pg/mL)	3967 (2556–7148)^a,b^	2508 (1217–6053)*	4323 (3367–7358)	3446 (2611–4049)*	7323 (5432–9097)	1136 (547–3984)	1597 (1281–2781)
CSF pNf-H (ng/mL)	0.43 (0.21–1.25)	0.54 (0.29–1.45)*	0.41 (0.16–0.95)	0.45 (0.22–0.95)*	0.04 (0.04–0.33)	0.33 (0.04–1.36)	0.47 (0.19–1.43)

Values are presented as median (interquartile range) unless otherwise indicated

Gender and MMSE were analyzed with a Chi-squared test

Kruskal-Wallis test with post-hoc Dunn’s correction was used to compare (full) dementia groups and controls

Significant differences (*p* < 0.05) are indicated: ^a^ compared to Alzheimer’s disease; ^b^ compared to controls

Pairwise Mann-Whitney *U* tests were used to compare FTLD subgroups

Significant differences (*p* < 0.05) are indicated: * compared to FTLD-*GRN*; ^†^ compared to FTLD-tau

*Aβ* amyloid-beta, *CSF* cerebrospinal fluid, *FTLD* frontotemporal lobar degeneration, *LP* lumbar puncture, *Nf-L* neurofilament light chain, *MMSE* Mini-Mental State Examination, *N/A* not applicable, *pNf-H* phosphorylated neurofilament heavy chain, *p-tau*_*181*_ phosphorylated tau, *t-tau* total tau

#### Progranulin

**Fig. 1:**
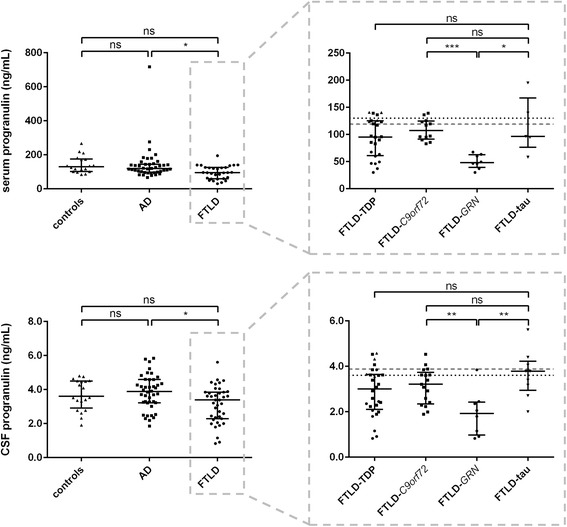
Dot plots of progranulin levels in all different (sub)groups. Values are presented as median and interquartile range. Left panels compare controls (triangles), Alzheimer’s disease (AD; squares), and frontotemporal lobar degeneration (FTLD; circles) dementia groups. Right panels compare FTLD subgroups: FTLD-TDP (triangles, squares (representing FTLD-*C9orf72* symptomatic mutation carriers), and circles (representing FTLD-*GRN* symptomatic mutation carriers)), and FTLD-tau (downward triangles)). The dotted line represents the median control level, and the dashed line represents the median AD level. ***p < 0.001, **p < 0.01, *p < 0.05. CSF cerebrospinal fluid, ns not significant

The FTLD group had significantly lower progranulin levels compared with AD and controls (serum, *p* < 0.01; CSF, *p* < 0.05). This effect was mainly driven by the FTLD-*GRN* patients, who had the lowest serum and CSF progranulin levels of all groups (Fig. 1); significances disappeared when these patients were excluded. The separation of FTLD-*GRN* carriers and FTLD patients without *GRN* mutations was achieved at a cut-off level of 75.3 ng/mL in serum (91% sensitivity, 100% specificity) and at 2.52 ng/mL in CSF (81% sensitivity, 88% specificity).

#### AD biomarker panel

**Fig. 2:**
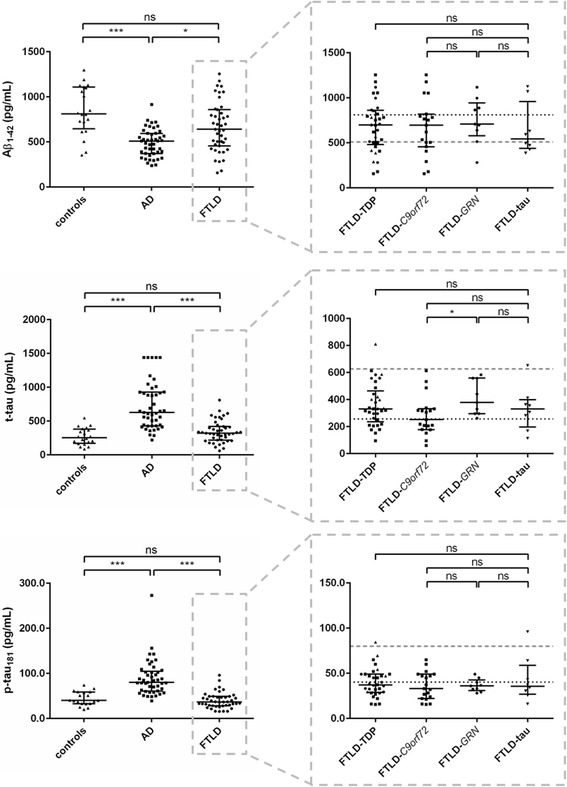
Dot plots of AD CSF biomarkers in all different (sub)groups. Values are presented as median and interquartile range. Left panels compare controls (triangles), Alzheimer’s disease (AD; squares), and frontotemporal lobar degeneration (FTLD; circles) dementia groups. Right panels compare FTLD subgroups: FTLD-TDP (triangles, squares (representing FTLD-*C9orf72* symptomatic mutation carriers), and circles (representing FTLD-*GRN* symptomatic mutation carriers)), and FTLD-tau (downward triangles)). The dotted line represents the median control level, and the dashed line represents the median AD level. ***p < 0.001, **p < 0.01, *p < 0.05. Aβ amyloid-beta, ns not significant, p-tau_181_ phosphorylated tau, t-tau total tau

There were no significant differences in t-tau or p-tau_181_ levels when comparing FTLD patients and controls. Levels of Aβ_1–42_ were normal for the main part of the controls, while covering a wide range from normal to abnormal values in the FTLD group (Fig. 2). Part of this variability could be explained by amyloid-β copathology as A2-C1, A2-C2, A3-C2, or A3-C3 scores (representing Thal (A) and CERAD (C) scores in Montine criteria [34–36]) were given in 5/14 of patients with abnormal Aβ_1–42_ values below the pathological cut-off for AD (< 638.5 pg/mL [29]) and in no patients with normal Aβ_1–42_ values above the pathological cut-off. There was no association with age at LP, age at onset, or MMSE score in these patients. Additionally, there was an effect of *APOE* ε4 carrier status on Aβ_1–42_ levels in FTLD (*p* = 0.036).

Within the FTLD group, no significant differences were found for Aβ_1–42_ or p-tau_181_, but there was a difference in t-tau levels, being significantly higher in FTLD-*GRN* patients (Fig. 2). In these patients t-tau was also significantly higher than in controls (*p* < 0.05). There was no significant difference in p-tau_181_/t-tau ratio between FTLD-TDP and FTLD-tau groups (*p* = 0.29). While not significantly different from the other FTLD subgroups, Aβ_1–42_ levels in the FTLD-tau group were quite low and did not significantly differ from the AD group (*p* = 0.20).

#### Neurofilaments

**Fig. 3:**
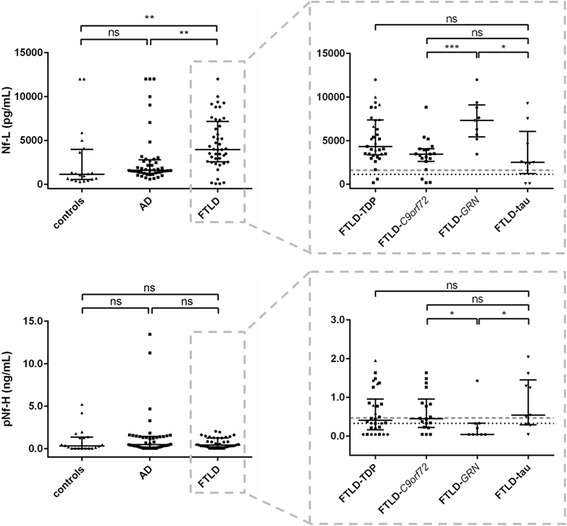
Dot plots of neurofilament protein levels in all different (sub)groups. Values are presented as median and interquartile range. Left panels compare controls (triangles), Alzheimer’s disease (AD; squares), and frontotemporal lobar degeneration (FTLD; circles) dementia groups. Right panels compare FTLD subgroups: FTLD-TDP (triangles, squares (representing FTLD-*C9orf72* symptomatic mutation carriers), and circles (representing FTLD-*GRN* symptomatic mutation carriers)), and FTLD-tau (downward triangles)). The dotted line represents the median control level, and the dashed line represents the median AD level. ***p < 0.001, **p < 0.01, *p < 0.05. Nf-L neurofilament light chain, ns not significant, pNF-H phosphorylated neurofilament heavy chain

Nf-L was higher in the FTLD group in comparison with AD or controls, with post-hoc pairwise comparisons between FTLD and AD or FTLD and controls both being significant (*p* < 0.01). There was no significant difference in pNf-H levels between the diagnostic groups (Fig. 3).

With regard to FTLD subgroups, pNf-H was significantly higher in FTLD-*C9orf72* and FTLD-tau compared with FTLD-*GRN* (both *p* < 0.05). Conversely, Nf-L was significantly higher in FTLD-*GRN* compared with FTLD-*C9orf72* and FTLD-tau (*p* < 0.001 and *p* < 0.05, respectively; Fig. 3). The difference between the entire FTLD-TDP subgroup compared to FTLD-tau did not reach significance (*p* = 0.052). While all FTLD subgroups had higher Nf-L levels than controls, this difference was only significant for FTLD-*GRN* and FTLD-TDP (both *p* < 0.001). The same results were found when comparing the different FTLD subgroups with AD patients, but with an additional significance for FTLD-*C9orf72* (*p* < 0.05).

#### Correlations between biomarkers

Interestingly, serum and CSF progranulin levels correlated only in the FTLD cohort. Specifically, this correlation was seen in both FTLD-tau (ρ = 0.900, *p* < 0.05, *n* = 5) and FTLD-TDP (ρ = 0.765, *p* < 0.001, *n* = 22), but in the latter group this was limited to FTLD-*GRN* (ρ = 0.821, *p* < 0.05, *n* = 7) as the correlation was absent in FTLD-*C9orf72* (ρ = 0.127, *p* = 0.71, *n* = 11). A remarkable correlation was found between the progranulin serum/progranulin CSF ratio and Nf-L levels in the *GRN* mutation carriers (ρ = 0.929, *p* < 0.01).

Biomarker levels of p-tau_181_ and t-tau correlated strongly in controls, AD, and FTLD groups (controls, ρ = 0.701; AD, ρ = 0.887; FTLD, ρ = 0.769; all *p* < 0.001). Values of pNf-H and Nf-L were significantly correlated in controls and AD groups but not in FTLD (controls, ρ = 0.641; AD, ρ = 0.346; both *p* < 0.05). A correlation could be found for Nf-L and the p-tau_181_/t-tau ratio in all groups between both markers for neurodegeneration.

### Diagnostic accuracy

To differentiate between FTLD and AD, a reference logistic regression model generated with the established AD biomarkers achieved an AUC of 0.942 (95% CI 0.891–0.981). The only investigated biomarker that added value to this model was Nf-L, resulting in an AUC of 0.948 (95% CI 0.900–0.984) but this improvement was not significant (*p* = 0.30). The addition of age as a covariate did not change the result of the logistic model (AUC 0.951, 95% CI 0.908–0.956; *p* = 0.70).

For the differentiation between FTLD and controls, Aβ_1–42_ and Nf-L were the best predictors with an AUC of 0.881 (95% CI 0.761–0.971). The addition of age as a covariate did not significantly alter this model, gaining an AUC of 0.879 (95% CI 0.759–0.970; *p* = 0.66).

## Discussion

The lack of biomarkers specific for FTLD is a limiting factor in both clinical and research settings as it severely hampers the diagnostic certainty for this disorder. As such, the aim of this study was to validate both proven and high-ranking candidate CSF biomarkers to improve (differential) diagnosis of FTLD. A major advantage of this study was the availability of a large cohort of definite FTLD patients with genetic and/or neuropathological confirmation. Indeed, discrepancies between publications describing potential biomarkers for FTLD are largely due to the fact that clinical diagnosis is used to define patient groups. Conversely, in publications that used a definite diagnosis, patient groups were usually small, and more data are required to support their findings.

### Progranulin

In this study we confirm the use of decreased progranulin levels in either serum or CSF as a marker for mutation status in FTLD-*GRN* patients. No evidence was found that progranulin levels in CSF were significantly altered in FTLD patients without *GRN* mutations.

There are contradicting results about the correlation of blood and CSF progranulin levels in control subjects, being either absent [37, 38] or present [16, 17]. We did not find a significant correlation in either controls or AD patients, but CSF and serum progranulin levels did correlate very strongly in our FTLD-*GRN* subgroup (ρ = 0.821). This has only been looked at in one other study so far and, while the correlation was also significant, it was not as strong (Pearson’s *r* = 0.54 [38]). In our entire cohort with both serum and CSF available (*n* = 87), about 20.7% of CSF progranulin variability could be explained by serum progranulin variability (ρ^2^ = 0.207). This is considerably higher than previous studies which found that plasma progranulin variability only explained 6.2% or 13.1% of CSF progranulin variability, respectively [16, 17]. However, these studies did not include *GRN* mutation carriers in their analysis, and when we exclude these subjects the Spearman’s ρ^2^ was indeed only 0.099 (i.e., 9.9% explained) in our entire noncarrier group (*n* = 80). Another difference is the use of the nonparametric Spearman’s correlation coefficient in this study instead of the Pearson’s correlation coefficient. Using the latter in our entire cohort we found an *r*^2^ of only 0.105 (i.e., 10.5% explained). Focusing on the subgroup of *GRN* mutation carriers with available serum and CSF (*n* = 7), we found that 67.4% of CSF progranulin variability could be explained by serum progranulin variability. This is much higher than the 29% that was reported recently [38]. Our findings indicate that progranulin might thus be assessed in serum instead of CSF, but only for the FTLD-*GRN* group. As patient cohorts were small in both studies, further investigation of this relationship is definitely warranted.

### AD biomarkers

In the entire FTLD group there was a marked variability in Aβ_1–42_ and t-tau levels. The alteration in Aβ_1–42_ was not related to specific FTLD subtypes, although it was more prominent in FTLD-tau. In some patients, a low level of Aβ_1–42_ could be attributed to an Aβ copathology and/or *APOE* ε4 carrier status, but it might also be connected to individual variation in the Aβ production process [39].

The variability of t-tau was an interesting finding, as the main purpose in using the established AD markers was to see if tau proteins had an additional value in FTLD subtypes, in particular for FTLD-tau. However, neither t-tau nor p-tau_181_ were significantly different between FTLD-TDP and FTLD-tau, or between FTLD patients and controls. We found that the neuropathological subtypes also had comparable p-tau_181_/t-tau ratios (although slightly lower in FTLD-TDP than FTLD-tau). This contradicts previous studies that stated that the ratio was significantly lower in FTLD-TDP [21, 22, 40]. It should be noted that, while they gained significance, differences in p-tau_181_/t-tau ratios in these previous studies were also very small. Additionally, there has been much discussion about the (unspecific) pathological changes that would result in a differential p-tau_181_/t-tau ratio between FTLD-TDP and FTLD-tau [40]. In fact, the ratio has been contradictorily decreased either by lower p-tau_181_ or higher t-tau levels [21, 41]. As such, and together with our data in pathologically confirmed patients, we doubt that the p-tau_181_/t-tau ratio will be valuable in the diagnosis of FTLD subgroups. Interestingly, when looking at genetic subtypes of FTLD-TDP, it appears that t-tau is specifically elevated in patients carrying a *GRN* mutation since their levels were significantly higher than those of *C9orf72* mutation carriers, at least explaining some of the variability in the FTLD group (see also the paragraph ‘Differences within FTLD subtypes’ below).

### Neurofilament proteins

With regard to neurofilament levels in controls, AD, and FTLD subjects, there was only a significant increase in Nf-L in FTLD. To evaluate the influence of amyotrophic lateral sclerosis (ALS), which is characterized by high Nf-L levels [42], statistical analysis was performed without FTLD-TDP patients with associated ALS (*n* = 5) which had no effect on the results. In comparison to AD we observed a 2.5-fold increase in FTLD, which is in concordance with other studies that found a two- to threefold concentration of Nf-L in FTLD compared with AD [26, 40, 43]. Some older studies did not find a significant difference between the two types of dementias, but these studies had the limitation of assessing clinical FTLD patients only and separating the AD cohort based on age of onset [25, 44]. We observed more than a threefold increase in Nf-L levels in FTLD compared with controls, which is again in agreement with other studies [25, 26, 43, 45]. As for pNf-H, previous studies with clinical patient cohorts have reported differences in pNf-H levels between FTLD and controls, but differentiation with AD has not been possible [25, 44, 46]. By confirming the lack of significant differences in definite FTLD patients, we conclude that focus should remain on Nf-L rather than pNf-H.

While we established that Nf-L was higher in FTLD in general, we investigated if there was an association with a specific underlying pathology and found a trend towards increased Nf-L values in FTLD-TDP compared with FTLD-tau (*p* = 0.052). As the same relation has gained significance in other studies [26, 40] the small number of FTLD-tau patients in our study likely limited our findings. In the genetic subgroups, a significant difference for Nf-L was found between *GRN* and *C9orf72* mutation carriers (see also the paragraph ‘Differences within FTLD subtypes’ below).

### Combination of biomarkers for differential diagnosis of FTLD

In this study, the diagnostic accuracy obtained with the established AD biomarkers was used as a reference for comparison with the other investigated biomarkers. It should be noted that, while the use of AD CSF biomarkers is known to increase diagnostic certainty, the clinical diagnosis of AD patients remains suboptimal which is a limitation of this study and has likely influenced differential diagnostic accuracy. Our results show that only p-tau_181_ and Aβ_1–42_ were necessary to differentiate between AD and FTLD, confirming previous results in definite FTLD patients [47, 48]. The discriminative power could be improved on with the inclusion of Nf-L. While diagnostic accuracies of AD markers on their own and Nf-L on its own have been described previously, only one study performed a joint ROC analysis and also reported an improved diagnostic accuracy when using the combination of Aβ_1–42_, p-tau_181_, and Nf-L in comparison with Aβ_1–42_ and p-tau_181_ alone [25]. Nf-L also showed added value in the differentiation between FTLD and controls, confirming its usefulness as a marker for FTLD [40, 45].

### Differences within FTLD subtypes: genetics and/or neuropathology

As stated above, both CSF levels of t-tau and Nf-L were markedly higher in FTLD-*GRN* patients compared with other FTLD subgroups (Table 1). There are limited data available with which to compare these findings, but for Nf-L at least it has been described that levels are significantly higher in FTLD-*GRN* than in FTLD-*C9orf72* and FTLD-*MAPT* [45]. Regarding tau, one study in contrast reported lower levels of t-tau and p-tau_181_ in FTLD patients with a *GRN* mutation than FTLD patients without a *GRN* mutation, although they were not expecting this result [49]. Indeed, there is evidence that FTLD-*GRN* patients would specifically have more generalized brain atrophy, more white matter lesions, a faster rate of neurodegeneration, and a resulting shorter disease duration [50–53]. Our findings certainly support this notion, as the common feature of both t-tau and Nf-L proteins is that they are markers for general neurodegeneration.

## Conclusions

This study adds to the available biomarker data by validating different markers in an FTLD cohort with definite diagnoses. Nf-L has an added diagnostic value for the differentiation between FTLD and AD, and between FTLD and controls. The value of progranulin as a biomarker is shown to be limited to those patients with a *GRN* mutation. Established AD biomarkers are very variable in FTLD and their diagnostic value is based on the exclusion of AD pathology. None of the evaluated biomarkers is able to differentiate between pathological subtypes of FTLD. Also, the relation between CSF and serum levels of progranulin remains ambiguous, but at least appears to be present in *GRN* mutation carriers. Within this FTLD subgroup, elevated CSF levels of t-tau and Nf-L indicate a more severe neurodegeneration.

## Abbreviations

AD: Alzheimer’s disease
ALS: Amyotrophic lateral sclerosis
AUC: Area under the curve
Aβ_1–42_: Amyloid-beta of 42 amino acids
CI: Confidence interval
CSF: Cerebrospinal fluid
ELISA: Enzyme-linked immunosorbent assay
FTLD: Frontotemporal lobar degeneration
IBB: Institute Born-Bunge
LP: Lumbar puncture
MMSE: Mini-Mental State Examination
Nf-H: Neurofilament heavy chain
Nf-L: Neurofilament light chain
p-tau_181_: Tau protein phosphorylated at threonine 181
ROC: Receiver operating characteristic
TDP-43: Transactive response DNA-binding protein of 43 kDa
t-tau: Total tau protein
